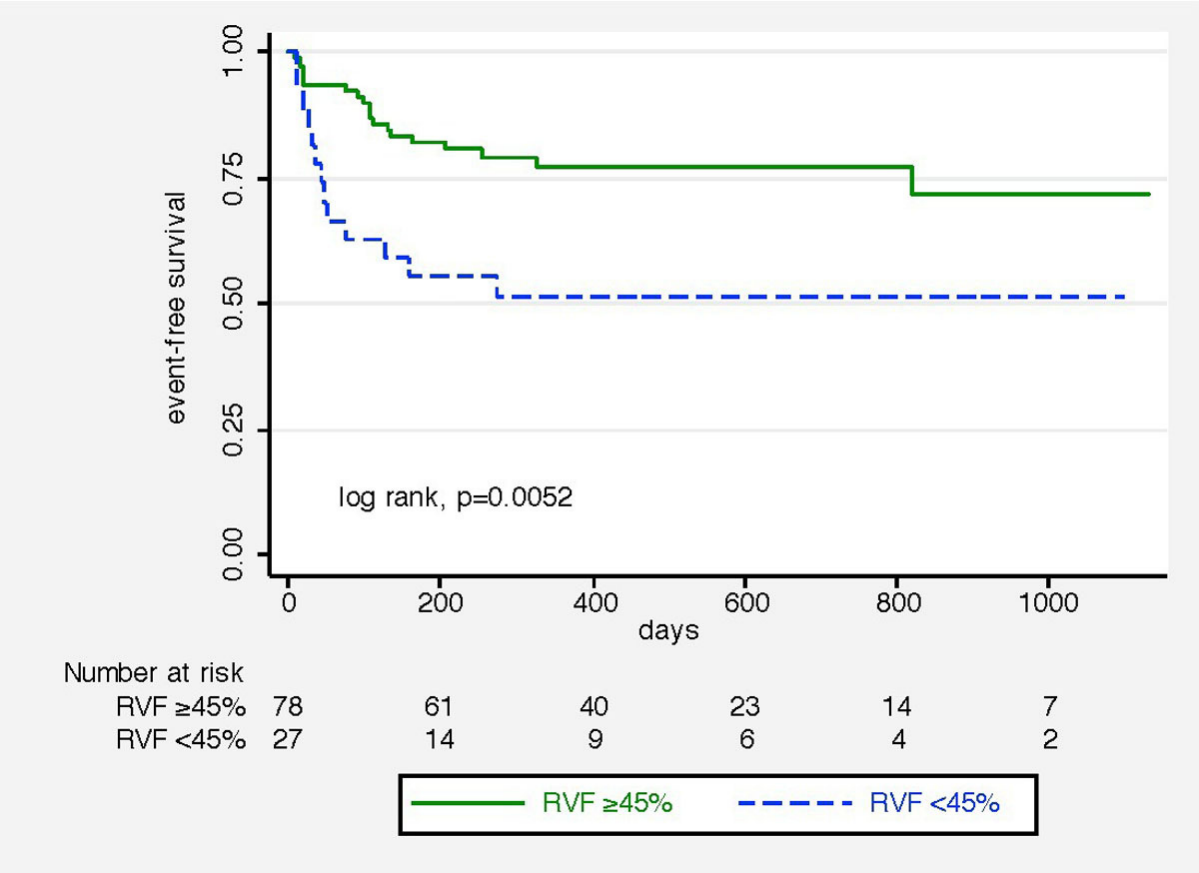# Prevalence and prognostic significance of right ventricular systolic dysfunction in heart failure with preserved ejection fraction. Insights from a cardiac magnetic resonance imaging study

**DOI:** 10.1186/1532-429X-17-S1-O33

**Published:** 2015-02-03

**Authors:** Stefan Aschauer, Caroline Tufaro, Andreas Kammerlander, Alina F Bachmann, Diana Bondermann, Julia Mascherbauer

**Affiliations:** 1 Cardiology, Medical University of Vienna, Vienna, Austria

## Background

Cardiac magnetic resonance imaging (CMR) is the gold-standard technique for the assessment of right ventricular function. Recent data indicate that right ventricular ejection fraction (RVEF) <45% by CMR is a strong predictor of outcome in patients with dilated cardiomyopathy. However, the prognostic significance of RVEF in heart failure with preserved ejection fraction (HFpEF) is unknown.

## Methods

Between December 2010 and September 2013 we prospectively enrolled 105 HFpEF patients. At baseline, all patients underwent CMR imaging in addition to invasive and non-invasive testing. Right ventricular systolic dysfunction (RVSD), defined as an RV ejection fraction <45%, was present in 27 (25.71%) patients. Patients were followed for 434 ± 325 days, during which 31 had a cardiac event (hospitalization for heart failure and/or death for cardiac reason).

## Results

By univariate Cox analysis RVSD (p=0.007), NYHA class III+IV (p=0.006), 6-minute-walking-distance (p<0.001), diabetes (p<0.001) and mean pulmonary artery pressures (p<0.001) were significantly associated with outcome. By multivariable analysis only RVSD (HR 3.23, CI 1.50 - 6.92, p=0.003), diabetes (HR 3.51, CI 1.47 - 8.40 p= 0.005) and mean pulmonary artery pressure (HR 1.04, CI 1.00 - .108 p= 0.002) remained significant predictors of cardiac events. In addition, patients with RVSD presented with significantly higher resting heart rate (p= 0.022), more advanced NYHA functional class (p= 0.016) and shorter 6-minute-walking-distance (t-test p= 0.016). By Kaplan Meier analysis, outcome was significantly worse in patients with RVSD (log rank, p=0.0052).

## Conclusions

Although HFpEF is considered a disease of the left ventricle respective imaging parameters are not related with outcome. In contrast, RVSD by CMR is significantly associated with mortality and clinical status in these patients, and can be used for risk-stratification.

## Funding

None.

**Figure 1:**